# Editorial: The COVID-19 pandemic crisis: The implication for work-life/family balance and gender inequalities

**DOI:** 10.3389/fsoc.2022.1075705

**Published:** 2022-11-21

**Authors:** Isabella Crespi, Vera Lomazzi

**Affiliations:** ^1^Department of Education, Cultural Heritage and Tourism, University of Macerata, Macerata, Italy; ^2^Department of Human and Social Sciences, University of Bergamo, Bergamo, Italy

**Keywords:** work-life/family balance, gender equality, unpaid care, wellbeing, Europe, pandemic, COVID-19

The need for action for Sustainable Development pursuing gender equality (Objective 5 in the UN 2030 Agenda) finds, among the multiple areas of commitment, a direct link with the domain of work-life balance, a topical issue for gender equality. In this direction, the European Union issued a new Work-life Balance Directive in 2019, aimed at promoting gender equality through increased female economic participation and a fairer share of unpaid care responsibilities between men and women.

Despite the relevance given to this topic, several factors still damper the progress toward greater gender equality and jeopardize the potential impact of national and transnational policies, including those in the European gender mainstreaming framework (Ahrens et al., 2018; Lomazzi and Crespi, 2019). For example, the deterioration of economic conditions because of the crisis (Karamessini and Rubery, 2013), austerity policies (Anastasiou et al., 2015), and the institutionalization of far-right, populist, and Euro-skepticism movements (Meret and Siim, 2013; Akkerman, 2015) can contribute to a traditional backlash.

In this scenario of permanent crisis, the pandemic might represent a further threat to gender equality: Pre-existing gender inequalities (EIGE, 2019) further increase also because of the gendered implications of the increased challenges to work-life/family balance, as papers belonging to this Research Topic show.

In this line, mental health and wellbeing during the pandemic are important issues. In the paper by Burn et al., the significant changes in workplace practices as social distancing requirements caused by the COVID-19 pandemic are analyzed using a cross-sectional survey of European working women to explore their experiences during the initial stages of the pandemic and the association with the symptoms of depression. A higher prevalence of depression symptoms is found among women working from home, compared to those traveling to a workplace.

Looking at potential protective factors against such risks, Lakshmi and Oinam's paper discusses the impact of yoga on the work-life balance of working women during the COVID-19 pandemic as a protective factor of wellbeing.

Living arrangements experiences during the pandemic and how private living situations shaped individuals' experiences of this crisis also play a role in the impact on wellbeing. In their paper, Langenkamp, Cano and Czymara analyze how experiences and concerns vary across living arrangements from a social disadvantages perspective. Using data from a web survey fielded in Germany, the authors analyze how people belonging to different household structures (living alone, shared living without children, living with a partner and children, and single parents) experienced and coped with the pandemic situation during the first wave of the pandemic, showing higher risks for single mothers of experiencing care-related worries.

Some of the papers included in this Research topic concentrate on the gender divide particularly.

Hartner-Tiefenthaler et al. report that women carried the major burden of additional housework in families in Austria. While gender affected perceptions of working-from-home during the pandemic independently from children, the presence of children seemed to increase the existing burden, especially for women. A critical aspect emerging from this research is that working-from-home can generally be seen as an enabler to reduce work-life/family conflict for both women and men, but bears different challenges based on the contextual family and personal situation.

The paper by Izquierdo-Useros et al. studied the impact of COVID-19 lockdowns on scientific activity, domestic and caregiving tasks, and psychological status. Again, the results revealed differences in scientific performance by gender. While male researchers participated in many scientific activities for career development, female researchers performed more invisible scientific tasks, including peer review or outreach activities. Results spot a disproportionate impact of COVID-19 lockdowns on female scientific career development and urge for equity measures to mitigate the consequences of an increase in the gender gap in biomedical sciences.

Moreno-Mínguez and Ortega-Gaspar focused on the COVID-19 pandemic that has intensified existing imbalances in family-work responsibilities in general and the ICT gender gap. COVID-19 has exposed these imbalances, highlighting the need for new narratives and laws that encourage gender equality. Post-COVID-19 scenarios thus present an opportunity for reflection and progress on Spanish family policy. From this perspective, the authors suggest that the paradigm of work-family conflict should be re-evaluated, including changes in the expectations and practices around work-family balance based on family diversity, job insecurity, the technological revolution, and new masculinities.

As suggested in this last paper, the issue of measures and policies to mitigate the impact of COVID 19 on inequalities is an important one. The paper by Daly focuses right on this point. In particular, she analyses the policy responses to both care for young children and frail, ill or disabled adults and develops an understanding of care as a welfare-related activity focused on practices and resources oriented to meeting care-related needs in the European context. The main focus is on how European countries responded to the 2020 pandemic, especially regarding the types of care need that were recognized, the resources committed, the actors/agency that was supported or taken for granted, and the values underpinning the responses. Daly's main result shows that while care assumed a strong place in the public rhetoric, this was not reflected in greater public resourcing of care for young children or long-term care. Instead, care for children was re-familiarized and long-term care was under-resourced and relegated to a secondary position; both were in many ways rendered further dependent on the private agency of individuals.

The papers collected in this Research Topic contribute to a solid and multidimensional assessment of the impact of the COVID-19 pandemic on work-life/family balance and the implications for gender equality. As pointed out in the scientific debate on this topic, the pandemic enlightened European states' difficulties in protecting from the “old” social risks and providing innovative responses to the “new” social risks and needs emerging from the new situation. This results in cumulative challenges that build on pre-existing inequality conditions. Among the many consequences of the pandemic, we have seen that not only gender inequality increased concerning the division of paid work and unpaid care between women and men, but, as the papers in this Research Topic show, the gendered impact on mental health, wellbeing, productivity, and work performance risk amplifying gender inequalities when policies are unable to support individuals and families in their multiple roles as workers and caregivers.

## Author contributions

All authors listed have made a substantial, direct, and intellectual contribution to the work and approved it for publication.

## Conflict of interest

The authors declare that the research was conducted in the absence of any commercial or financial relationships that could be construed as a potential conflict of interest.

## Publisher's note

All claims expressed in this article are solely those of the authors and do not necessarily represent those of their affiliated organizations, or those of the publisher, the editors and the reviewers. Any product that may be evaluated in this article, or claim that may be made by its manufacturer, is not guaranteed or endorsed by the publisher.
